# High affinity of β-amyloid proteins to cerebral capillaries: implications in chronic lead exposure-induced neurotoxicity in rats

**DOI:** 10.1186/s12987-023-00432-5

**Published:** 2023-05-01

**Authors:** Luke L. Liu, Xiaoli Shen, Huiying Gu, Gang Zhao, Yansheng Du, Wei Zheng

**Affiliations:** 1 School of Health Sciences, Purdue University, 550 Stadium Mall Drive, HAMP-1273, West Lafayette, IN 47907 USA; 2 School of Public Health, Qingdao University, Qingdao, China; 3 Department of Neurology, Indiana University School of Medicine, Indianapolis, IN USA; 4 Department of Medical Biology, School of Basic Medical Sciences, Hubei University of Chinese Medicine, Wuhan, China

**Keywords:** Lead (Pb), Amyloid beta (Aβ), Cerebral vasculature, Low density lipoprotein receptor-related protein 1 (LRP1)

## Abstract

Lead (Pb) is a known environmental risk factor in the etiology of Alzheimer’s disease (AD). The existing reports suggest that Pb exposure increases beta-amyloid (Aβ) levels in brain tissues and cerebrospinal fluid (CSF) and facilitates the formation of amyloid plaques, which is a pathological hallmark for AD. Pb exposure has long been associated with cerebral vasculature injury. Yet it remained unclear if Pb exposure caused excessive Ab buildup in cerebral vasculature, which may damage the blood–brain barrier and cause abnormal Ab accumulation. This study was designed to investigate the impact of chronic Pb exposure on Aβ accumulation in cerebral capillary and the expression of low-density lipoprotein receptor protein-1 (LRP1), a critical Aβ transporter, in brain capillary and parenchyma. Sprague–Dawley rats received daily oral gavage at doses of 0, 14 (low-dose), and 27 (high-dose) mg Pb/kg as Pb acetate, 5 d/wk, for 4 or 8 wks. At the end of Pb exposure, a solution containing Aβ_40_ was infused into the brain via the cannulated internal carotid artery. Data by ELISA showed a strikingly high affinity of Ab to cerebral vasculature, which was approximately 7–14 times higher than that to the parenchymal fractions collected from control brains. Pb exposure further aggravated the Aβ accumulation in cerebral vasculature in a dose-dependent manner. Western blot analyses revealed that Pb exposure decreased LRP1 expression in cortical capillaries and hippocampal parenchyma. Immunohistochemistry (IHC) studies further revealed a disrupted distribution of LRP1 alongside hippocampal vasculature accompanied with a decreased expression in hippocampal neurons by Pb exposure. Taken together, the current study demonstrated that the cerebral vasculature naturally possessed a high affinity to Aβ present in circulating blood. Pb exposure significantly increased Aβ accumulation in cerebral vasculature; such an increased Aβ accumulation was due partly to the diminished expression of LRP1 in response to Pb in tested brain regions. Perceivably, Pb-facilitated Ab aggravation in cerebral vasculature may contribute to Pb-associated amyloid alterations.

**Supplementary Information:**

The online version contains supplementary material available at 10.1186/s12987-023-00432-5.

## Highlights

1. Affinity of Aβ_40_ to cerebral vasculature was exceptionally high.
2. Pb exposure exacerbated Aβ_40_ accumulation in cerebral vasculature.
3. LRP1 expression was disrupted by Pb in brain parenchyma and vasculature.

**Supplementary Information:**

The online version contains supplementary material available at 10.1186/s12987-023-00432-5.

## Introduction

Aggregation of beta-amyloid (Ab) peptides in the brain extracellular space to form the insoluble plaques is the hallmark of Alzheimer’s disease (AD). Excess accumulation of Ab in the cortex and hippocampus initiates the detrimental cellular cascades leading to the progressive degeneration of neurons and the ensuing cognitive deficits [33]. In AD brains, Ab is found in the cerebrospinal fluid (CSF) circulating in brain ventricles, in the interstitial fluid (ISF) bathing neurons and glial cells and in cerebral vasculature [42, 49, 51, 61]. Elevated Ab levels in brain extracellular space may result from an increased production of Ab peptides due primarily to genetic predisposition and/or a decreased clearance of Ab from the brain.

Sporadic cases account for over 90% of AD, suggesting a substantial contribution of environmental factors such as exposure to neurotoxic metal lead (Pb). Indeed, human studies have shown strong associations between lifetime Pb exposure and progressive cognitive declines and brain structural damages [19, 32, 48]. The presence of a high level of Pb in diffuse neurofibrillary tangles, a form of presenile dementia, has been shown in 10 AD cases compared with 9 controls [24]. The formation of senile plaques is evident in a patient exposed to Pb at age of 2.3 years old with a confirmed Pb encephalopathy and died at 42 [36]. Mechanistic studies using animal models also suggest causal associations of early-life Pb exposure with epigenetic alterations, amyloid plaque formation, and late-onset AD [67]. Research by this group has demonstrated that chronic Pb exposure accelerates the Aβ plaque formation and increases cognitive deficits in an AD transgenic mouse model (Tg-SWDI) [20, 22]. Interestingly, the Tg-SWDI mice possess a relatively high ratio of Aβ_40_ to Aβ_42_ and exhibit substantial cerebral amyloid angiopathy (CAA); such pathologies were further aggravated by chronic Pb exposure, leading to an even higher level of brain Aβ_40_ that is typically seen in CAA [20, 22].

Literature reports also provide evidence to support a linkage between Pb exposure and cerebral vasculature injury. For example, cerebral vasculature shows a higher propensity to accumulate Pb than do other brain cell types [56, 58, 74]. Pb exposure causes the damage to the cerebral vasculature that constitutes the blood–brain barrier (BBB) [25, 63, 74]. In addition, Pb exposure targes both heart and vascular smooth muscle and causes hypertension [41, 43, 59, 60]. Noticeably, “seeding” of exogenous Aβ from the peripheral circulation likely triggers an extensive Ab aggregation in the brain [2, 28]. Indeed, amyloid species exist in the blood circulation, with the concentrations increased in AD patients [26]. Considering the combined effects of Pb exposure, brain microvascular injury, and the ensuing amyloid accumulation, it is reasonable to postulate that Pb exposure may cause abnormal Aβ accumulation in cerebral vasculature, which may contribute to Pb-induced AD pathogenesis. However, the question as to whether Pb exposure increased Aβ_40_ buildup in cerebral vasculature has never been investigated.

The homeostasis of Aβ in brain extracellular fluids is maintained by a number of Aβ binding and transporting proteins. In general, the receptor for advanced glycation endproducts (RAGE) is believed to be a primary transporter at the BBB that transports Aβ from the blood to brain parenchyma (i.e., influx) [11] and the low-density lipoprotein receptor-related protein 1 (LRP1) serves as the main transporter of Aβ from brain to the blood (i.e., efflux) [13, 39]. Previous studies from this lab have established that Pb exposure increases Aβ levels in the choroid plexus, a barrier between the blood and CSF; this increase is concurrent with a decreased expression of LRP1 [22] and the abnormal intracellular trafficking of LRP1 in choroidal epithelia [3, 52]. Nonetheless, it remained unknown if Pb overload in brain capillaries would alter LRP1 expression and therefore affect Aβ efflux in the BBB.

The main purposes of the present study were to investigate whether and how in vitro and in vivo Pb exposure affected the accumulation of Aβ in the cerebral vasculature. We used in situ brain infusion technique to deliver Aβ_40_ molecules directly into the brain via the internal carotid artery, followed by a capillary depletion procedure to separate the cerebral vasculature from parenchyma, allowing to quantify Aβ concentrations in cerebral vasculature and brain parenchymal fractions from various brain regions. We further analyzed the expression of LRP1 in regional vascular and parenchymal fractions with a particular focus on hippocampus. The results may shed light on the mechanism by which Pb exposure causes AD pathology.

## Material and methods

### Materials

Chemical reagents and assay kits were purchased from the following sources: lead acetate trihydrate (PbAc_2_·3H_2_O), sodium pyruvate, calcium chloride (CaCl_2_), Dextran-70, HEPES, 2-mercaptoethanol, phenylmethylsulfonyl fluoride (PMSF), poly-acrylamide and tetramethyl-ethylenediamine (TEMED) from Sigma Chemicals (St Louis, MO); ultrapure nitric acid from VWR international (Chicago, IL); protease inhibitor cocktail from Calbiochem (San Diego, CA); Tris-base, glycine, sodium dodecyl sulfate (SDS), 2X Laemmli sample buffer, Triton X-100, and Clarity Western ECL substrate from Bio-Rad (Hercules, CA); human Aβ_40_ recombinant peptides, human Aβ_40_ ELISA kit from Invitrogen, goat anti-rabbit secondary antibody conjugated with Alexa Fluor 488, goat anti-rat secondary antibody conjugated with Cyanine5, goat anti-rabbit secondary antibody conjugated with horseradish peroxidase (HRP), and goat anti-mouse secondary antibody conjugated with HRP from Invitrogen (Waltham, MA); and Dextran (75,000) from Spectrum Chemicals (Gardena, CA). Sources of primary antibodies and dilution factors are listed in Table 1. All reagents were of analytical grade, HPLC grade, or the best available pharmaceutical grade.

**Table 1:** Primary antibodies and dilutions used for Western Blot and IHC studies

Target	Dilution (application)	Host species	Source and identifier
LRP1	1:1000 (WB); 1:200 (IHC)	Rabbit	Abcam ab92544
Beta-actin	1:2000 (WB)	Mouse	Sigma-Aldrich A5316
CD31	1:500 (IHC)	Rat	Invitrogen MA1-40074

### Animals

Mice were initially used for concept-proving preliminary in vitro studies as described in “Preparation of cerebral capillary fraction and in vitro Aβ40 affinity study” section. Most of the experiments in this report were performed using rats for technical feasibility of in situ brain perfusion and large yields of capillary fraction. C57BL/6 mice (3 months old) and Sprague Dawley rats (10 weeks old) were purchased from Harlan Sprague Dawley Inc. (Indianapolis, IN). Literature data suggest that the animal strains used in this study with a wild type background develop no Aβ pathologies at the time of our experiments [4, 38], indicating a minimal level of endogenous Aβ. Upon arrival, animals were housed in a temperature-controlled room under a 12-h light/12-h dark cycle and allowed to acclimate for one week prior to experimentation. Animals had free access to deionized water and pellet rat chow (Teklad Dlobal 18% Protein Rodent Diet, 2018s; Envigo) ad libitum. This study was conducted in compliance with standard animal use and practice and was approved by Purdue Animal Care and Use Committee (PUCAC No. 1112000526).

### Preparation of cerebral capillary fraction and in vitro Aβ_40_ affinity study

**Fig. 1:**
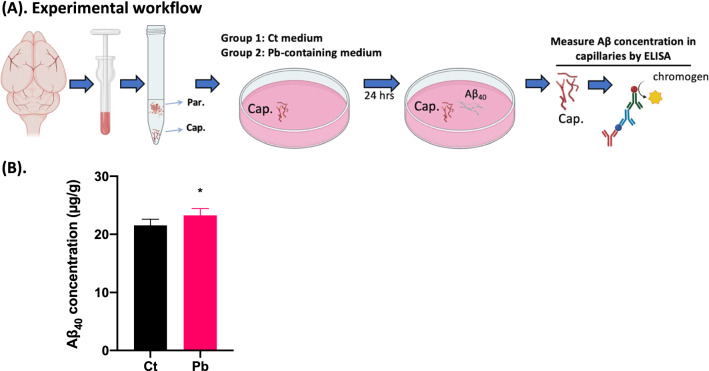
Increased Aβ binding to cerebral capillaries following in vitro Pb exposure. **A** Graphical illustration of the experimental workflow to assess Aβ_40_ affinity as affected by Pb exposure using freshly separated cerebral capillaries in vitro*.* **B** ELISA quantification of Aβ_40_ concentration in control or Pb-pretreated cerebral capillaries. Data represent mean ± SD, n = 6; *p < 0.05. *Par.* parenchyma fraction, *Cap.* capillary fraction

Under deep anesthesia, animals were transcardially perfused with ice-cold PBS and the brains were carefully extracted. Cerebral vasculature was separated using a well-established “capillary depletion” method in this lab [7, 12, 14] (as illustrated in Fig. 1A). Briefly, ice-chilled brain samples were weighted and transferred to a 1-ml glass tissue grinder (Wheaton, Millville, NJ, followed by addition of 3 volumes of ice-cold homogenization buffer [HB solution (g/mL); brain weight/HB volume, which consisted of the following chemicals with their final concentrations: HEPES 10 mmol/L, NaCl 141 mmol/L, KCl 4 mmol/L, MgSO_4_ 1.0 mmol/L, NaH_2_PO_4_ 1.0 mmol/L, CaCl_2_ 2.5 mmol/L, and glucose 10 mmol/L (pH 7.4)]. The entire brain from mice or separate brain regional tissues from rats were homogenized by eight strokes, followed by addition of another 4 volumes of 30% Dextran-70 solution in a final ratio of 1:3:4 (brain: HB: dextran-70). The mixture was then homogenized for three additional strokes. After centrifugation at 5400×*g* for 15 min at 4 °C, the supernatant (capillary-depleted parenchyma) and the pellet (capillary-enriched fraction) were separated carefully. The presence of networks of brain vessels in the pellet and a vasculature-depleted supernatant were confirmed by light microscopy. Notably, although this method represents an effective approach for vasculature-parenchyma separation, isolated vasculature can still somewhat contain, besides endothelial cells, other cell types such as vascular smooth muscle cells and pericytes [40].

To prove the concept of Pb altering Aβ_40_ affinity to cerebral capillaries, an in vitro study to incubate cerebral capillary with Aβ_40_ was conducted. Freshly separated cerebral capillaries were incubated in the control HBSS buffer (supplemented with 0.5% BSA) without Pb or in the HBSS containing Pb concentration at 10 µM for 24 h, after which Aβ_40_ was added to the culture medium at a final concentration of 1 μM. The incubation continued for 30 min at room temperature with gentle rotation. Upon completion of incubation with Aβ_40_, the capillary samples were washed with HBSS three times to remove the unbound Aβ_40_. Capillaries were then lysed in 5 M guanidine-HCl for 1 h at room temperature and quantified for Aβ_40_ concentrations using enzyme-linked immunosorbent assay (ELISA) (detailed in “Quantification of Aβ40 in both parenchyma and capillary by ELISA” section).

### In vivo Pb exposure and in situ brain perfusion

PbAc_2_·3H_2_O was dissolved in sterile saline for oral gavage. Rats were exposed to Pb by oral gavage at doses of 14 and 27 mg Pb/kg, as low- (LD-Pb) and high-dose (HD-Pb) group, respectively (the control received the same volume of saline), once daily, 5 days/week, for 4 or 8 consecutive weeks. This Pb exposure dosing regimen was used based on our prior studies, in which the blood Pb levels in Pb-exposed animals were similar to those observed in occupational workers [21, 52].

Following Pb exposure for 4 or 8 weeks, in situ brain perfusion was conducted to investigate how Pb exposure affected the Aβ_40_ affinity to cerebral capillaries and brain parenchyma. This technique has been routinely used by this laboratory for studies such as trace element metal copper (Cu) and Aβ peptide uptake by BBB and blood-CSF barrier (BCB) [14, 31, 52]. Briefly, 24 h after last gavage, rats were anesthetized by ketamine/xylazine (75:10 mg/kg, ip) and placed on a heating pad in a supine position. The right common carotid artery was isolated, and a small cut was made. A polyethylene catheter (PE-10) tubing (prefilled with Ringer’s solution and connected with a peristaltic pump through a 3-way stopcock) was then carefully inserted into the artery toward the brain. The inserted tubing was then fixed by a surgical suture, followed by a ligation of the external carotid artery to ensure the flow of the perfusate entering exclusively to the internal carotid artery, which supplies blood for the brain.

The brain was perfused with Ringer’s solution (warmed to 37 °C and continuously gassed with 95% O_2_/5% CO_2_) by a Mini-Pump (VWR, Radnor, PA) at 9 mL/min. The second syringe pump (pre-connected with the 3-way stopcock) was subsequently turned on to infuse Ringer’s solution containing Aβ_40_ at 25 µg/mL with a flowrate of 1 mL/min. The total flow rate of perfusion by two pumps was 10 mL/min with the infusate (Aβ_40_) concentration at 2.5 µg/mL. Considering the high flowrate (10 mL/min) and Pb’s cytosol accumulation (sequestration by intracellular glutathione or metallothionein), it is anticipated that Aβ_40_ in the perfusate has little direct contact with Pb; hence, any alteration in Aβ_40_ affinity to capillaries is considered due primarily to the interaction between Aβ_40_ molecules and endothelial cells through surface binding and intracellular uptake. The in-situ brain perfusion with Aβ_40_ lasted 2 min. To prevent blood recirculation, the left ventricle of the heart was cut upon the start of the perfusion. After 2-min Aβ_40_ perfusion, the pump with Aβ_40_ solution was shut off and the pump with only Ringer’s solution was left on for 1 min to remove Aβ_40_ adsorbed to the luminal surface.

At the end of brain perfusion, the brains were extracted from the skull; hippocampus, frontal cortex and the rest of brain tissues were dissected for capillary-parenchyma separation as described in “Preparation of cerebral capillary fraction and in vitro Aβ40 affinity study” section. Concentrations of Aβ_40_ in the collected vascular and parenchyma fractions were measured by ELISA following manufacturer’s instructions.

### Quantification of Aβ_40_ in both parenchyma and capillary by ELISA

Total proteins of brain capillary and parenchyma were extracted with a buffer containing 50 mmol/L Tris–HCl, 150 mmol/L NaCl, 1% Nonidet P-40, 0.5% sodium deoxycholate, 1 mmol/L EDTA, and a protease inhibitor cocktail. The protein concentrations were determined using the Bradford assay. Levels of Aβ_40_ were assayed by sandwich ELISA as previously described per manufacturer’s instructions [22, 52]. Briefly, the protein extracts of capillary and parenchymal fractions of hippocampus, cortex and the rest of brain were incubated in 96-well ELISA plates that had been coated with Aβ_40_-capturing antibodies. Subsequently, Aβ_40_-detector antibodies were added and incubated for 3 h at room temperature. Following sufficient washes to remove the unbound antibodies, IgG HRP solution was added to bind the detector antibodies; the wells were covered and incubated for 30 min at room temperature. Through thorough washes, chromogen solutions were added to each well and continued to incubate at room temperature for 30 min under dark. After addition of stop solutions, the plates were read for absorbance at 450 nm. The concentration of Aβ_40_ in the tissues was reported as ng/g of total protein.

### Western blot (WB)

WB assay was performed as previously described [31]. Briefly, the extracted protein samples from brain regional capillaries and parenchyma were diluted with sample buffer to the final protein concentrations of appropriately 2 mg/mL. Protein samples were mixed with 2× Laemmli sample buffer and boiled for 5 min. Samples (10 µL containing 10 µg protein) were then loaded on the 8 + 15% dual-layer tris–glycine SDS–polyacrylamide gels, electrophoresed and transferred to PVDF membranes. The membranes were blocked with 5% dry milk (fat free) in Tris-buffered saline with 0.1% Tween 20 (TBST) and incubated overnight at 4 °C with the primary antibodies. Following sufficient washes by TBST, PVDF membranes were further incubated with HRP-conjugated secondary antibodies at room temperature for 1 h. The colorimetric signals on the membranes were developed using ECL Western Blotting substrate by Molecular Imager and captured by ChemiDoc XRS + Software (Bio-Rad, Hercules, CA). Beta-actin was used as an internal control. The band intensities were quantified using ImageJ and were reported as relative expression levels to beta-actin.

### Immunohistochemistry (IHC)

At the end of in vivo Pb exposure, rat brain samples were prepared for IHC characterization of LRP1 expression as previously described [31]. Briefly, 4% paraformaldehyde (PFA)-perfused brain samples were further fixed in this fixative solution under 4 °C overnight. Following dehydration in 30% sucrose solution, brains samples were treated into 40-µm brain slices by a microtome, and then preserved in cryopreservation medium (30% sucrose, 1% polyvinylpyrrolidone, 30% ethylene glycol in 0.1 M phosphate buffer) under − 20 °C. Upon IHC staining, brain slices with regions of interest were collected and washed with PBS 3 times. The subsequent blocking was performed by incubating brain slices in PBST containing 5% normal goat serum (NGS) and 0.3% TritonX-100 for 1 h at room temperature. Blocked slices were then incubated with primary antibodies targeting LRP1 and CD31 overnight at 4 °C. Following 3× PBST washes, brain slices were further incubated with fluorophore-conjugated secondary antibodies (1:500) for 1 h at room temperature prevented from light. Following 3× PBST washes, stained slices were mounted onto microscope slides with mounting medium containing DAPI. Negative control staining was performed using the same procedures except for the primary antibody incubation step: following blocking with NGS, slices directly incubated with secondary antibodies and DAPI (image provided in Additional file 1: Fig. S1). The IHC images were captured by Nikon A1Rsi Confocal system. To quantitatively assess expression of LRP1 in response to Pb exposure, fluorescent intensity of LRP1 in anatomically matched regions was quantified using ImageJ in known neuron-rich regions for neuronal and in CD31(+) cells for endothelial assessment. LRP1 fluorescent intensity was normalized against the control group measurements for statistical analysis.

### Determination of Pb concentrations by atomic absorption spectrophotometry (AAS)

Pb concentrations in blood or tissues were quantified by AAS as described in our previous publications [52, 75]. Samples (200 mg of wet weight) were digested in a MARSX press microwave-accelerated reaction system with 0.20 mL ultrapure concentrated nitric acid at 200 °C for 4 h. Blood samples (200 mL) were digested with nitric acid in the oven at 55 °C overnight. An Agilent Technologies 200 Series SpectrAA with a GTA 120 graphite tube atomizer was used to quantify Pb concentrations. The standard curves were prepared daily at concentrations of 0, 4, 8, 12, 16, and 20 μg/L with correlation coefficient of r^2^ = 0.9869. The detection limit was 1.35 ng Pb/mL of assay solution. The intra-day and inter-day precisions of the method were 1.5% and 2.9%, respectively.

### Statistical analysis

All data are presented as mean ± standard deviation (SD). Statistical analyses of the differences between control and Pb-exposed group(s) were carried out by Student’s t-test or one-way ANOVA with post hoc comparisons by the Dunnett’s test. All the statistical analyses were conducted using GraphPad Prism (San Diego, CA). The differences between two means were considered significant if *p* values were equal or less than 0.05.

## Results

### In vitro Pb exposure increased Aβ_40_ binding to the cerebral capillaries

To test the concept that Pb increased the binding of amyloid species to cerebral vasculature, we conducted a preliminary study by preparing cerebral capillaries freshly isolated from normal mouse brains and pre-incubating the capillary preparation with Pb for 24 h, followed by incubation with Aβ_40_ in the medium (Fig. 1A). Data by ELISA quantification revealed that Pb treatment significantly increased the Aβ_40_ concentrations in the cerebral capillaries by 7.88%, as compared to controls (*p* < 0.05) (Fig. 1B). This in vitro finding suggested that Pb exposure appeared likely to facilitate the Aβ accumulation in cerebral vasculature. This observation led to the following in vivo validation and characterization.

### In vivo chronic Pb exposure induced excessive Aβ_40_ affinity by cerebral capillaries

**Fig. 2:**
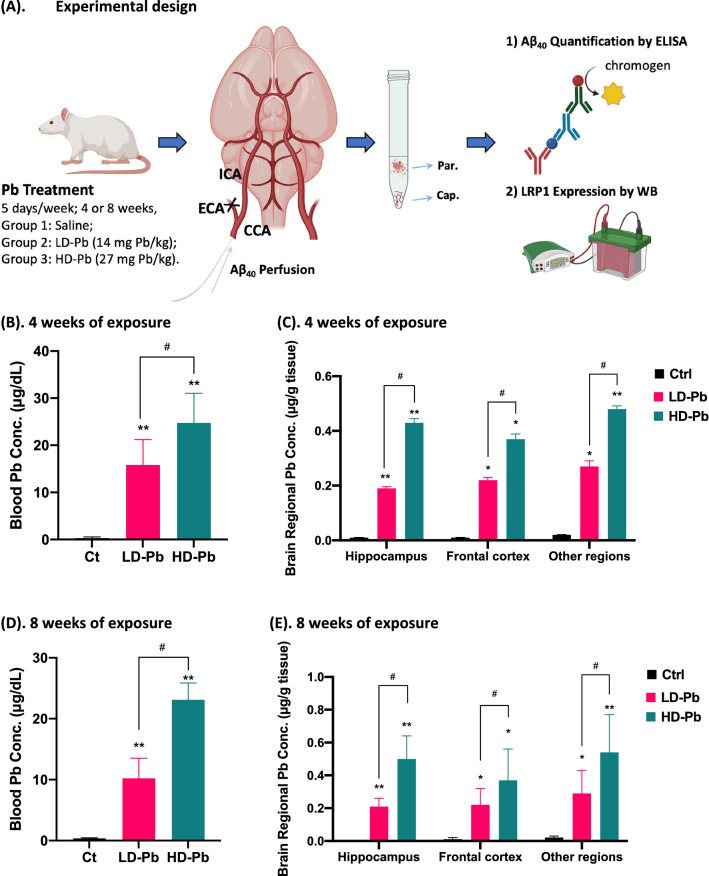
Experimental design of chronic in vivo Pb exposure and Pb levels in rat blood and selected brain tissues. **A** Graphical illustration of the experimental design to study the Aβ_40_ affinity to cerebral capillaries (cap.) and parenchyma (par.) via in situ brain perfusion technique and the effects of chronic Pb exposure. Capillary and parenchyma fractions of hippocampus, frontal cortex, and other brain regions were separated using a capillary depletion procedure for ELISA quantification of Aβ_40_ concentration; Western blot (WB) was performed to assess the LRP1 expression. **B**, **C** Blood Pb concentration and brain regional Pb concentration following 4 weeks of Pb exposure. **D**, **E** Blood Pb concentration and brain regional Pb concentration following 8 weeks of Pb exposure. Data represent mean ± SD, n = 4–8; *p < 0.05, **p < 0.01, as compared to the controls; ^#^*p* < 0.05, as compared to the low Pb exposure group. *ICA* internal carotid artery, *ECA* external carotid artery, *CCA* common carotid artery

In situ brain perfusion is a unique technique to study substance’s uptake en route from the cerebral vasculature to parenchyma, for its direct delivery of testing molecules into the cerebral artery with a subsequent quantitation of the partition of the testing molecule between blood vessels and parenchyma. In the current study, rats were chosen for surgical convenience and the large yield of capillary fraction. Figure 2A depicts the workflow of in vivo Pb exposure dose regimen, in situ brain perfusion, capillary/parenchyma separation, and assays used. Data by AAS demonstrated that the blood lead levels (BLLs) were 15–25 mg/dL and 10-23 mg/dL, in rats following 4 and 8 weeks of Pb oral gavage exposure, respectively (Fig. 2B, D). Correspondingly, Pb concentrations in hippocampus, frontal cortex and other regions were significantly increased in Pb-exposed animals (Fig. 2C, E).

**Fig. 3:**
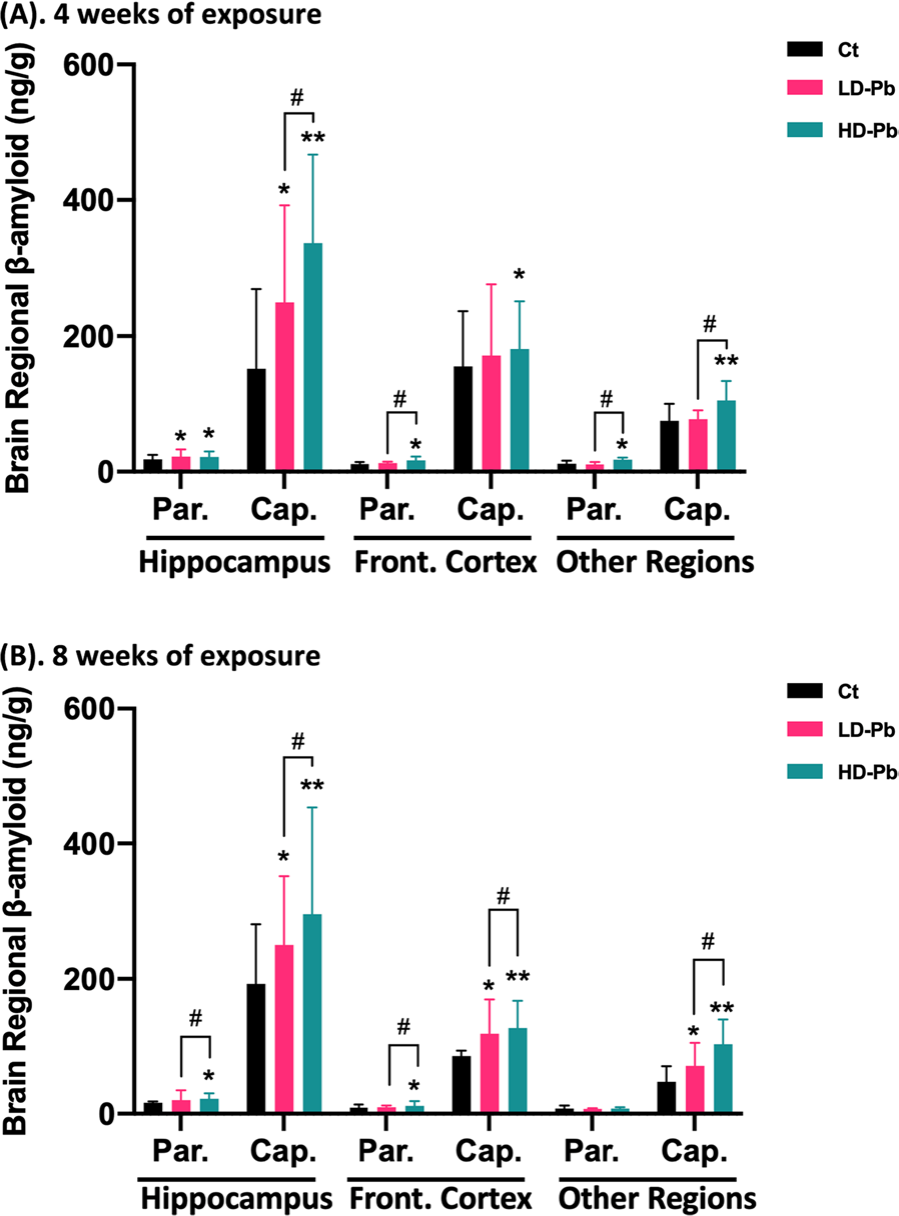
Increased Aβ binding to cerebral capillaries following chronic in vivo Pb exposure. **A**, **B** Quantification of Aβ_40_ concentration in cerebral parenchyma and capillaries following in situ brain perfusion of Aβ_40_. Parenchymal and vascular fractions of specific brain regions were sampled from rats exposed to Pb (14 mg or 28 mg Pb/kg) and controls for 4 or 8 weeks. Data represent mean ± SD, n = 4–8; *p < 0.05, **p < 0.01, as compared to the controls in specific brain fraction; ^#^*p* < 0.05 as compared to the low Pb exposure group in specific brain fraction. *Par.* parenchyma fraction, *Cap.* capillary fraction

On these Pb-exposed and control animals, we performed a 2-min in situ perfusion of Aβ_40_; ELISA was then used to quantify Aβ_40_ concentration in cerebral capillary and parenchyma fractions. Our data showed that under normal physiological condition, the average Aβ_40_ in normal hippocampal capillary from control rats without Pb exposure was 9.9 times higher than that in normal hippocampal parenchyma (*p* < 0.01). Similarly, the average Aβ_40_ in normal cortex capillary is 11.7 times higher than that in cortex parenchyma (*p* < 0.05) (Fig. 3A). Thus, it became apparent that the degree to which Aβ_40_ molecules bound to the cerebral capillaries, regardless of brain regions, far exceeded their binding to parenchyma (Fig. 3A, B), suggesting a naturally high binding affinity of Aβ_40_ to cerebral vasculature.

Chronic Pb exposure clearly increased Aβ_40_ accumulation in capillary and parenchymal fractions. Following 4-week Pb exposure, the Aβ_40_ levels in hippocampal capillaries were significantly increased by 65.3% (*p* < 0.05) and 122.9% (*p* < 0.01) in the low and high Pb exposure group, respectively, as compared to controls (Fig. 3A). The 8-week Pb exposure induced similar dose-dependent Aβ_40_ accumulation in hippocampal capillaries (Fig. 3B). In addition, Pb exacerbated Aβ_40_ accumulation in cerebral capillaries isolated from the frontal cortex, but to a degree less than that in hippocampus. For example, concentrations of Aβ_40_ in the frontal capillary, following 4 weeks of high-dose Pb exposure, was elevated by 17.0% (*p* < 0.05), but not in the low exposure group, as compared with controls. After 8 weeks of exposure, the Aβ_40_ levels in frontal cortex capillaries were increased by 38.8% and 48.2% in the low and high Pb exposed animals (*p* < 0.05), respectively, versus controls.

In contrast, the concentrations of Aβ_40_ detected in brain parenchyma were much lower than those in cerebral capillaries (Fig. 3A, B). Among tested brain regions, hippocampal parenchyma was most susceptible to Pb-elevated Aβ_40_ deposition. A 4-week Pb exposure increased Aβ_40_ detected in this fraction by 18–20% in both Pb-treated groups in comparison to controls (*p* < 0.05). Pb exposure for 8 weeks at the high level increased even more Aβ_40_ deposition in hippocampal parenchyma by 36.5% (*p* < 0.05), while the low-level exposure for 8 weeks did not alter Aβ_40_ levels. The parenchymal fractions collected from the frontal cortex and the rest brain regions showed the similar trend in accumulating Aβ_40_, following in vivo Pb exposure at low- and high-doses for 4 or 8 weeks (Fig. 3A, B).

Overall, the observations from our in situ perfusion study suggested that cerebral vasculature possessed an intrinsic high affinity to Aβ_40_ present in blood circulation; chronic Pb exposure seems likely to render the cerebral vasculature more venerable to Aβ_40_ accumulation.

### Pb exposure decreased LRP1 expression in specific fractions of hippocampus, frontal cortex, and other regions

**Fig. 4:**
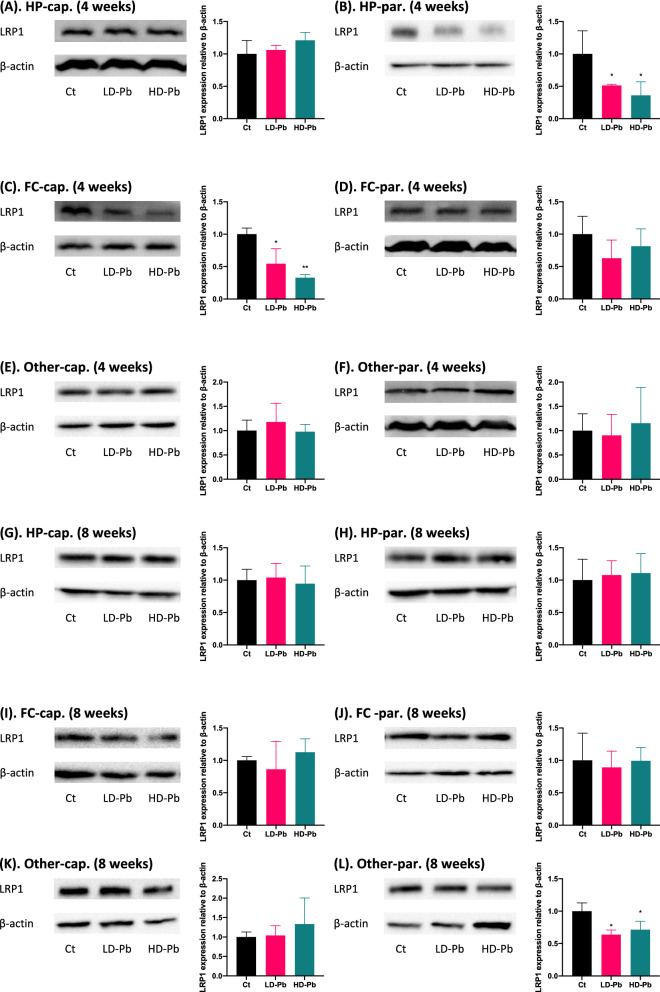
Altered LRP1 expression in brain capillary and parenchyma fractions following 4 or 8 weeks of in vivo Pb exposure in rats. **A**–**F** Effects of 4-week Pb exposure on LRP1 expression in hippocampal capillaries (**A**), hippocampal parenchyma (**B**), frontal cortex capillaries (**C**), frontal cortex parenchyma (**D**), capillaries of other brain regions (**E**), and parenchyma of other brain regions (**F**) by Western blot (WB). **G**–**L** Effects of 8-week Pb exposure on LRP1 expression in hippocampal capillaries (**G**), hippocampal parenchyma (**H**), frontal cortex capillaries (**I**), frontal cortex parenchyma (**J**), capillaries of other brain regions (**K**), and parenchyma of other brain regions (**L**) by WB. Data represent mean ± SD, n = 3; *p < 0.05 and **p < 0.01, as compared to the controls in specific brain fraction

To understand the mechanism by which Pb increased Aβ_40_ deposition in cerebral vasculature and brain parenchyma, we performed Western blot to determine LRP1 expression in collected brain factions with or without Pb exposure, since LRP1 is known to transport Aβ_40_ for its clearance. Following 4 weeks of exposure, LRP1 expression in hippocampal parenchyma was significantly decreased by 48.7% and 64.1% in the low and high Pb exposure groups, respectively, as compared to controls (*p* < 0.05) (Fig. 4B), whereas its expression in hippocampal capillary did not change (Fig. 4A). Interestingly, Pb exposure significantly reduced LRP1 expression in the capillaries of frontal cortex by 45.5% and 67.0% in the low- and high-exposure groups, respectively, in comparison to controls (*p* < 0.01) (Fig. 4C). However, in the rest of tested fractions, i.e., hippocampal capillaries, frontal cortex parenchyma, and both fractions in other brain regions, no significant LRP1 expression alterations were found following the 4-week exposure (Fig. 4A, D–F).

Exposure to Pb for 8 weeks did not significantly alter LRP1 expression in all tested fractions except for the parenchyma collected from the rest of brain samples. There was a significantly downregulated LRP1 expression in the parenchyma of other brain regions, by 36.5% and 25.6% in the low- and high-Pb exposure groups, respectively (*p* < 0.05) (Fig. 4L). These Western blot data suggested that Pb-induced decline in LRP1 expression may contribute, at least in part, to the increased accumulation of Aβ_40_ in cerebral vasculature and parenchyma.

### Pb exposure disrupted LRP1 expression in hippocampal neurons and frontal cortex capillaries

**Fig. 5:**
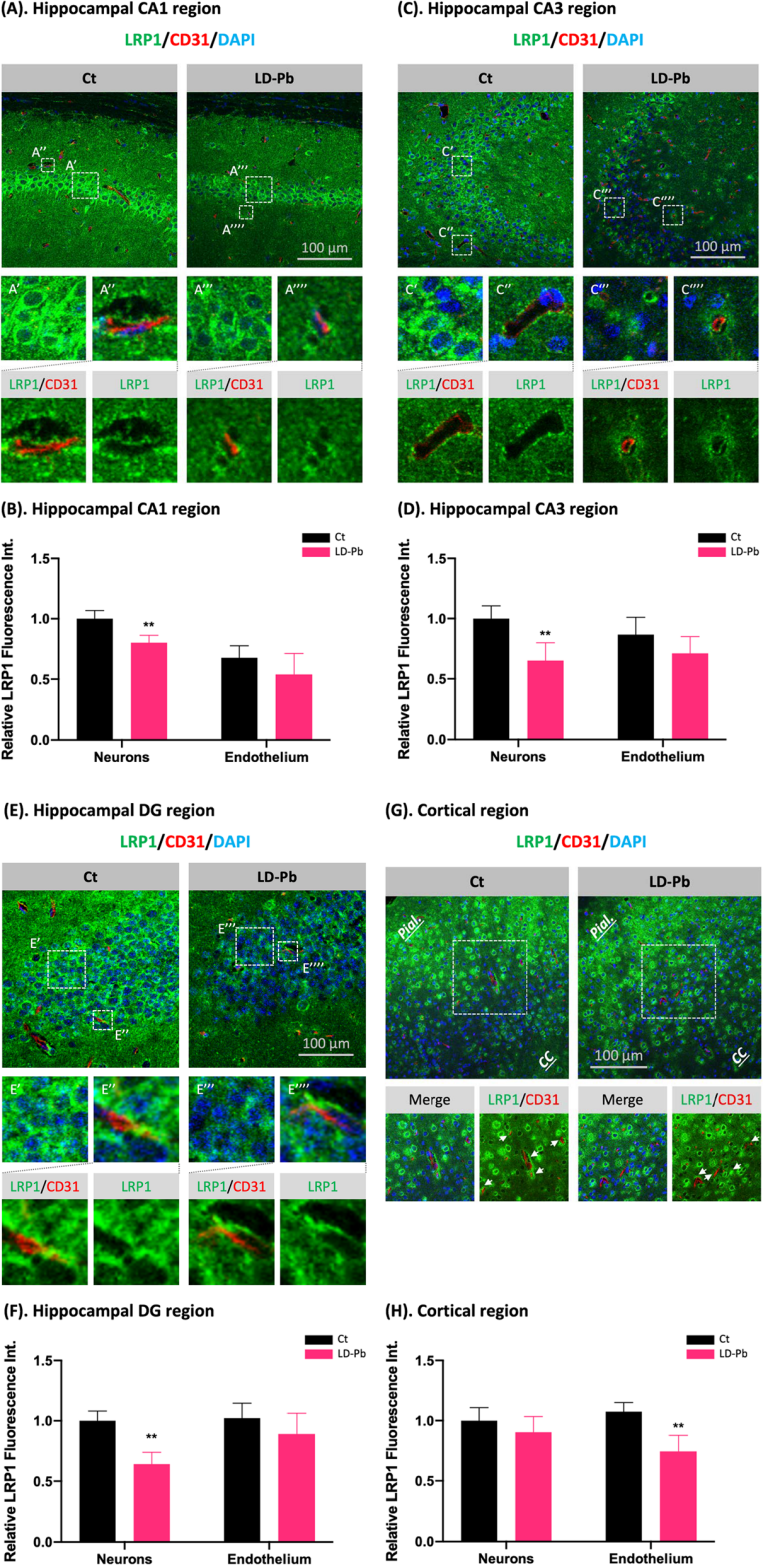
LRP1 expression in neurons and capillaries in hippocampal subfields and frontal cortex following 4 weeks of lose-dose in vivo Pb exposure. **A** IHC staining of hippocampal CA1 region with LRP1 and CD31. LRP1 expression in CA1 neurons (A′ and A‴) and capillaries (A″ and A⁗) were further magnified in panels below. **B** Quantification of LRP1 expression by fluorescent intensity in neurons and capillaries in hippocampal CA1 region. **C** IHC staining of hippocampal CA3 region with LRP1 and CD31. LRP1 expression in CA3 neurons (C′ and C‴) and capillaries (C″ and C⁗) were further magnified in panels below. **D** Quantification of LRP1 fluorescent intensity in neurons and capillaries in hippocampal CA3 region. **E** IHC staining of hippocampal dentate gyrus (DG) with LRP1 and CD31. LRP1 expression in DG neurons (E′ and E‴) and capillaries (E″ and E⁗) were further magnified in panels below. **F** Quantification of LRP1 expression by fluorescent intensity in neurons and capillaries in hippocampal DG. **G** IHC staining of frontal cortex region with LRP1 and CD31. Pial surface (Pial.) and corpus callosum (CC) were labeled to show LRP1 expression pattern. Selected regions were further magnified to show LRP1 expression in cortical neurons and capillaries as affected by Pb exposure. **H** Quantification of LRP1 expression by immunofluorescent intensity in neurons and capillaries in frontal cortex. Data represent mean ± SD, n = 4; **p < 0.01, as compared to the controls in the specific fraction

The CA1, CA3, dentate gyrus (DG) of hippocampus and frontal cortex are specifically susceptible to vascular and parenchymal Aβ deposition [46, 66]. To study the expression of LRP1 as affected by Pb exposure in these subregions, we used IHC to co-stain LRP1 with CD31, a marker for endothelial cells, in rats following 4 weeks of low-dose in vivo Pb exposure. In hippocampal CA1, neurons expressed a high level of LRP1 in the perinuclear regions in control brains (Fig. 5A, A′), an observation consistent with reports elsewhere [1, 69]. Pb exposure significantly decreased neuronal LRP1 expression by 19.8% (*p* < 0.01) (Fig. 5A‴, B). Labeled by CD31 for cerebral vasculature, signals by LRP1 in control brains mostly surrounded CD31(+) capillaries, with a spatial proximity observed between LRP1 and CD31 signals (Fig. 5A″). Pb exposure, however, caused a discontinuous LRP1 expression around CD31(+) endothelium and the dissociation of LRP1 from CD31 (Fig. 5A⁗). Nevertheless, the fluorescent intensity of LRP1 in CA1 capillaries was not significantly altered (Fig. 5B).

LRP1 in CA3 was highly enriched in neuronal cells in the control group, showing a strong perinuclear expression pattern (Fig. 5C′) similar to the CA1 region (Fig. 5A′). Pb exposure significantly decreased LRP1 expression in CA3 neuronal cells by 34.8% (Fig. 5C‴, D). Similar to the Pb-exposed CA1 region, LRP1 in CA3 capillaries of Pb-treated rats was discontinuous from CD31(+) endothelium (Fig. 5C⁗). Additionally, a LRP1-CD31 signal separation, i.e., a gap existing between LRP1 and CD31, was observed in Pb-exposed animals but rarely in controls. Yet, quantitative analyses of LRP1 fluorescent intensity in CA3 vasculature did not yield a significant difference (Fig. 5D).

LRP1 in the control DG was abundantly expressed by the local neuronal cells in the perinuclear areas (Fig. 5E′). Nevertheless, its expression was significantly reduced by 35.9% by Pb exposure (*p* < 0.01) (Fig. 5E‴, F). In DG vasculature, LRP1 expressed along CD31(+) capillaries remained to be continuous and strong in controls (Fig. 5E″); but Pb exposure seemed to disperse LRP1 signals around the CD31-expressing vascular cells (Fig. 5E⁗). However, quantitative analysis of LRP1 expression by the fluorescent intensity did not show significant changes in DG capillaries between groups (Fig. 5F).

Expression of LRP1 in the frontal cortex exhibited a gradient pattern with the signal intensity declining gradually from the dorsal area (closer to the pial surface; pial.) towards the ventral (the corpus callosum; CC) (Fig. 5G). In neuronal cells with the typical perinuclear distribution, LRP1 level was not significantly changed by Pb exposure (Fig. 5G, H). However, Pb exposure not only disrupted LRP1 distribution around the cortical vasculature but also decreased LRP1 expression (Fig. 5G, H).

Overall, through characterizing the LRP1 expression in specific brain subregions/fractions, our data showed that this Aβ_40_ transporting protein was adversely affected by Pb exposure.

## Discussion

Our findings revealed a strikingly high affinity of Aβ_40_ to cerebral capillaries, which was characterized by (1) a short time 2-min perfusion leading to Aβ_40_ accumulation; (2) the ability of the cerebral capillary in sequestering Aβ_40_ molecules far more than brain parenchyma in all tested brain regions; and (3) a high affinity of Aβ_40_ to the capillaries in hippocampus and frontal cortex. The exact mechanism by which Aβ_40_ accumulates in cerebral capillary is unknown. The initial binding of Aβ_40_ to cerebral capillaries, regardless of the subsequent biological events (transcytosis, exocytosis, or degradation), is largely dependent upon the cell-surface receptors; the subsequent receptor-mediated endocytosis occurs allowing for the Aβ_40_ uptake [30, 47]. Receptors critical to Aβ_40_ uptake such as RAGE, P-glycoprotein, integrins and scavenger receptors, etc., are known to express highly in the cerebral vasculature [68, 73]. This may explain partly of the high affinity of Aβ to cerebral endothelia following in situ perfusion. In addition, one of the pathways to eliminate cerebrovascular Aβ is via a perivascular drainage system transporting Aβ alongside the vascular wall. On the way entering this drainage system, Aβ molecules may polymerize into fibrils on vascular basement membrane by interacting with extracellular components, enabling excessive Aβ accumulation [70]. However, questions remain as to where Aβ molecules accumulate the surface of blood-facing endothelia, within the endothelial cytosol, and/or between basolateral endothelium and vascular smooth muscular layer, and whether cerebral endothelial cells possess the unique binding site(s) for Aβ molecules as compared to other peripheral endothelial cell types. Thus, our observation calls for more research in the future to uncover the mechanism underlying the high affinity of Aβ_40_ to the cerebral vasculature.

Pb exposure, either in vitro or in vivo, evidently increased the deposition of Aβ_40_ in cerebral capillary; both hippocampus and brain cortex appeared to sequester more Aβ_40_ than other brain regions. The status of Aβ in the cerebral vasculature is regulated by several coordinated processes, including the influx on the endothelial cell surface as discussed above, intracellular degradation, and/or the efflux or removal of Aβ by the BBB. Interestingly, cell-surface integrin, a molecule responsible for Aβ adhesion and uptake, can be upregulated in vasculature upon inflammation [35], a condition frequently reported in Pb-induced neurotoxicity [6]. A recent report also shows that a compromised BBB integrity, a typical neurotoxicity associated with Pb exposure [21, 56, 63], can aggravate the vascular Aβ_40_ accumulation [62]. It is, thus, highly possible that chronic Pb exposure in the current study may cause the damage to the BBB, which in turn exacerbates the Aβ-capillary binding. Since the high affinity of Aβ_40_ to cerebral capillaries is due to surface binding, intracellular uptake, or both, immunogold labeling of Aβ with electron microscopy is needed to explain this high affinity by characterizing the subcellular location of Aβ [54]. Alternatively, stimulated emission depletion (STED) microscopic technique, which provides superior imaging performance, can be utilized for the same purpose: colocalization of Aβ with endothelial luminal markers such as carbonic anhydrase IV (CA IV) [18] would differentiate the “binding” and “uptake”.

A defection in Aβ clearance represents another theory in pathological mechanism for AD [17, 29, 37, 44, 57, 65]. Routes to clear Aβ in the central nervous system involve receptor-mediated clearance through the BBB [10], interstitial fluid bulk flow through the perivascular/glymphatic system [27, 64], and absorption from the CSF through the choroid plexus [52]. In the BBB system, Aβ is mainly cleared by a LRP1-mediated efflux [29, 45, 55, p. 1]. Conditional knockdown of LRP1 in cerebral endothelial and vascular smooth muscle cells significantly accelerates the cognitive and memory deficits by exacerbating the formation of Aβ plaque and CAA in transgenic mice [29, 55]. The current study showed a Pb-induced decline of LRP1 expression, specifically in cortical capillaries, where the CAA often develops. In addition, in the Pb-exposed hippocampus, LRP1, which normally expressed continuously along the endothelia in controls, distributed abnormally in a discontinuous patten, suggesting a defective Aβ clearance [53, 55].

Noticeably also, reports in literature indicate that Aβ drainage occurs as interstitial fluid bulk flow via the perivascular space along the cerebral vasculature, and in the due process, aquaporin 4 (AQP4) transporter expressed on astrocytes determines the perivascular/glymphatic drainage [23]. Interestingly Pb exposure disrupts AQP4 expression and function [23]. The current study does not address Pb toxicity towards astrocytes and AQP4; yet this line of study deserves attention for further investigation. In addition, the choroid plexus (CP), despite its bidirectionality for Aβ transport, selectively effluxes Aβ from the CSF-facing to the blood-facing side [8]; however, Pb exposure impairs LRP1-associated Aβ efflux mechanism at the choroid plexus [22, 52, 69], which may contribute to the brain Aβ overload. The meningeal system also facilitates the Aβ efflux through the lymphatics vessels [9], but its susceptibility to Pb toxicity remains elusive. Overall, we propose that multiple overlapping or interactive Aβ removal mechanisms may collectively account for Pb-induced Ab accumulation in brain.

LRP1 is not solely expressed in cerebral vasculature but also present abundantly in neurons [34]; its presence promotes neuronal cell survival, axon growth, and neurite outgrowth [15, 16, 71]. Our data by IHC and WB indicated that in vivo Pb exposure specifically decreased LRP1 in hippocampal but not cortical neurons. This observation suggested that memory-related symptoms present in Pb-exposed populations or experimental animals could be, at least in part, caused by a decreased LRP1 expression in neuronal cells in these areas. Indeed, the use of pioglitazone, a peroxisome proliferator activated receptor-γ (PPAR-γ) agonist known to increase LRP1 in the brain, significantly ameliorated the learning and memory impairment in AD transgenic mice by upregulating neuronal LRP1 expression in hippocampal neurons [50]. Thus, the degree to which the decreased neuronal LRP1 expression may contribute to Pb-induced neurotoxicity including learning deficits deserves further testing.

The current study has two limitations. First, there was an apparent discrepancy between the increased hippocampal and cortical capillary Aβ accumulation and the altered LRP1 expression. While Aβ molecules continued to accumulate in brain capillary fractions after 8-week chronic Pb exposure, LRP1 expression was not significantly altered in these brain fractions except for the parenchyma fraction of the rest of brain. It is possible that Pb may dysregulate the expression of RAGE at BBB as it did in the blood–CSF barrier in the choroid plexus [52]. An increased RAGE in the choroid plexus following 8-week Pb exposure underlies an increased Ab uptake by the choroid plexus [52]. Similarly, increased Ab levels in the cerebral vasculature could be due to Pb-induced expression of RAGE in the BBB. Thus, the effect of chronic Pb exposure on many other Ab transporting proteins in the BBB deserves future exploration. In addition, Pb exposure is known to damage the cerebral vasculature [41, 43, 60]. Noticeably also, brain endothelial cells are regionally heterogenous, rendering them more diverse in response to Pb exposure [5], this regional heterogeneity is even greater in neuronal cells [72]. Hence, it is possible that the spatial cellular heterogeneity also contributed to LRP1 alterations in the frontal cortex capillaries and hippocampal parenchyma by Pb, but not in others.

This study, due to the limited resource in the original experimental design, did not explicitly examine the relationship between BBB injury and Ab accumulation. Thus, it is desirable to conduct histopathological experiments to verify how the altered BBB integrity following Pb exposure may facilitate binding of Ab molecules to cerebral vasculature in our future experiments.

In summary, the current study demonstrates that the cerebral vasculature naturally possesses a strikingly high affinity to Aβ present in circulating blood; Pb exposure, either in vitro or in vivo greatly increases Aβ accumulation in cerebral vasculature. Such an increased Aβ buildup is due partly to the diminished expression of an Aβ efflux carrier LRP1 in response to Pb in tested brain region and fractions. Mechanisms underlying these alterations and the relationship to the development Pb-induced AD deserve further experimental testing.

## Supplementary Information

**Additional file 1: Figure S1.** Negative control staining for immunohistochemistry experiments.

## Data Availability

Not applicable.
