# Supplementary material for: High affinity of β-amyloid proteins to cerebral capillaries: implications in chronic lead exposure-induced neurotoxicity in rats
Source: Fluids Barriers CNS. 2023 May 1;20:32. doi: 10.1186/s12987-023-00432-5 (PMC10150519; doi:10.1186/s12987-023-00432-5)
Supplement: Supplementary file 1 — Additional file 1: Figure S1. Negative control staining for immunohistochemistry experiments. [file 12987_2023_432_MOESM1_ESM.docx]

**Figure S1. Negative control staining for immunohistochemistry experiments.**


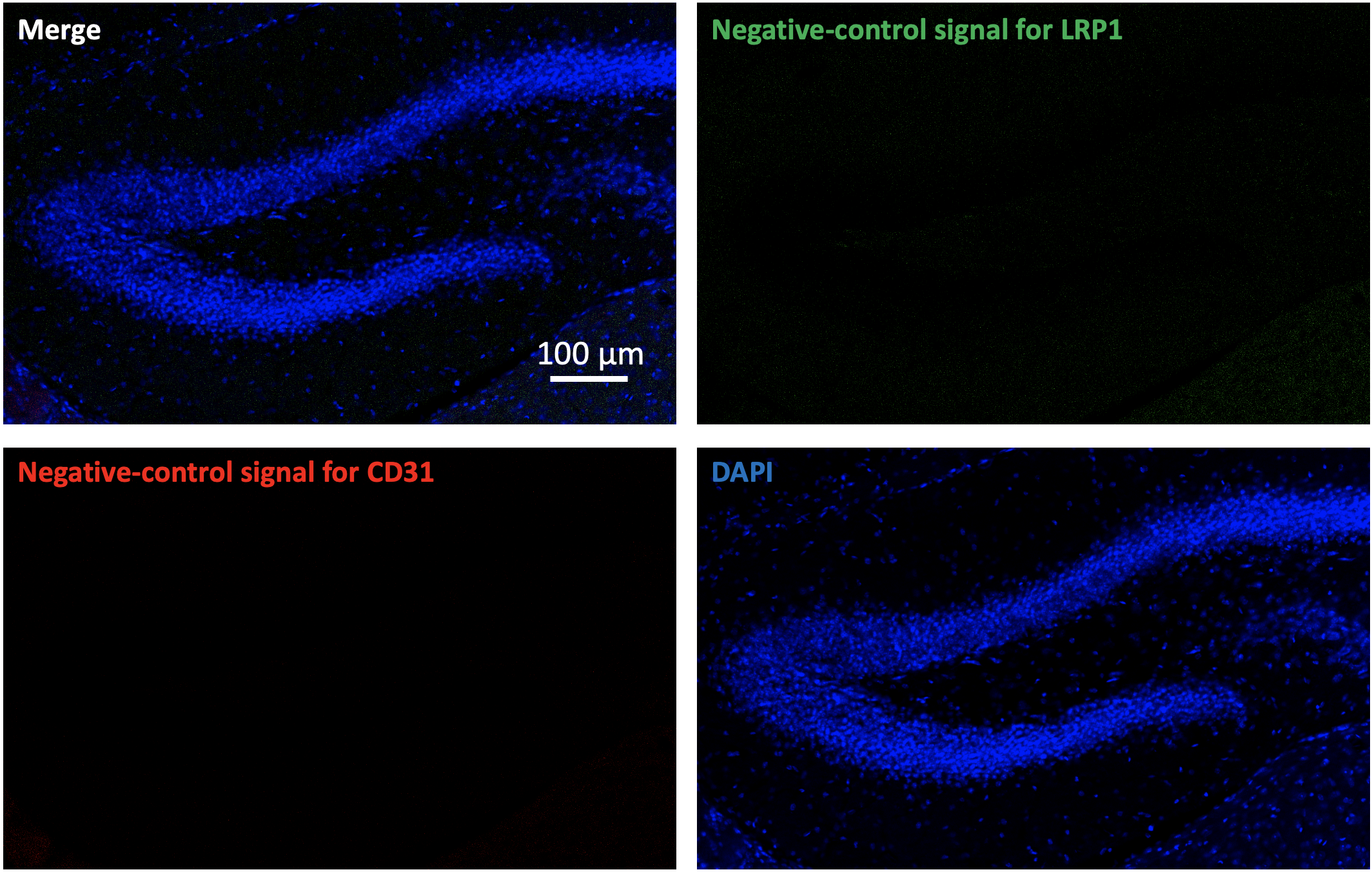


Negative-control staining was performed without primary antibodies with all the other steps the same. Scale bar = 100 µm.
